# METTL14-Mediated Inhibition of Apoptosis via the MAPK and PI3K/AKT Pathways Promotes *Chlamydia trachomatis* Reproduction

**DOI:** 10.3390/microorganisms14051025

**Published:** 2026-04-30

**Authors:** Wenbo Lei, Yewei Yang, Yating Wen, Hongrong Wu, Zhongyu Li

**Affiliations:** Institute of Pathogenic Biology, Hunan Provincial Key Laboratory for Special Pathogens Prevention and Control, Department of Clinical Laboratory Medicine, Institution of Microbiology and Infectious Diseases, The First Affiliated Hospital, Hengyang Medical School, University of South China, Hengyang 421001, China; leiwenbo2008@126.com (W.L.);

**Keywords:** *Chlamydia trachomatis*, m6A modification, METTL14, MAPK, PI3K/AKT, apoptosis

## Abstract

*Chlamydia trachomatis* has evolved sophisticated mechanisms to manipulate key host cell signaling pathways to facilitate its intracellular reproduction. N6-methyladenosine (m6A) in RNA is known to regulate various physiological and disease processes, and is also involved in the regulation of pathogenic and developmental processes in many pathogens. However, the specific impact of m6A modification on the intracellular growth of *C. trachomatis* remains poorly understood. In this study, our analysis of the m6A methylation profiles of host cell mRNAs following *C. trachomatis* infection revealed significant alterations in the distribution of m6A modifications, methylation motifs, and m6A-modified host target genes. We further demonstrate that chlamydial intracellular reproduction is mediated by the host methyltransferase-like (METTL) enzyme METTL14. Silencing METTL14 significantly reduced the reproduction efficiency of *C. trachomatis*. Mechanistically, *C. trachomatis* activates the Mitogen-Activated Protein Kinase (MAPK) and Phosphatidylinositol 3-kinase/Protein Kinase B (PI3K/AKT) signaling pathways through METTL14, thereby inhibiting host cell apoptosis and promoting intracellular bacterial reproduction. Collectively, these findings identify METTL14 as a key host factor for chlamydial intracellular reproduction, providing new mechanistic insights and potential targets for therapeutic intervention.

## 1. Introduction

*C. trachomatis* is an obligate intracellular bacterial pathogen that causes a range of diseases, including blinding trachoma, infertility, ectopic pregnancy, pelvic inflammatory disease and sexually transmitted diseases [1,2,3,4]. Notably, antibiotics treatment fail to completely eradicate the chlamydial infection in approximately 10% of cases [5]. Moreover, many infections are frequently asymptomatic if left untreated, resulting in progressive, irreversible pathological damage and posing a serious threat to human health.

During infection, *C. trachomatis* completes its entire developmental cycle within a membrane-bound compartment called an “inclusion”, which serves as a protective niche that shields the pathogen from host innate immune responses. Previous studies have shown that *C. trachomatis* modulates key host signaling pathways to alter cellular functions, including reprogramming cell death processes to facilitate its intracellular growth. However, the precise mechanisms governing the intracellular growth of *C. trachomatis* remain poorly understood.

m6A modification is the most common and widely studied internal modification among eukaryotic mRNAs. Increasing evidence has demonstrated that m6A plays a critical role in the intracellular replication and assembly, as well as in the evasion of host innate immunity, in a variety of pathogens, such as HIV [6,7,8], severe acute respiratory syndrome coronavirus clade 2 (SARS-CoV-2) [9,10], hepatitis B virus [11], herpes simplex virus 1 [12], hepatitis C virus [13], dengue virus [14], severe fever with thrombocytopenia syndrome virus [15], and *Mycobacterium tuberculosis* [16]. Nonetheless, whether and how *C. trachomatis* infection influences host m6A methylome dynamics remains largely unknown.

m6A methylation is catalyzed by the m6A methylation “writer” protein, the methyltransferase-like (METTL) enzymes METTL3 and METTL14 and the associated protein Wilms tumor 1-associated protein (WTAP) [17,18]. m6A modification is dynamically removed by two demethylases, fat mass and obesity-associated protein (FTO) and α-ketoglutarate-dependent dioxygenase ALKB homolog 5 (ALKBH5) [19,20]. Through these dynamic modifications, m6A influences diverse cellular processes, including apoptosis by regulating specific transcripts and key signaling pathways [21,22,23,24]. In particular, pathways such as MAPK and PI3K/AKT are central regulators of cell survival and have been shown to be targeted by *C. trachomatis* [25,26].

In this study, we focused on METTL14, which is dynamically upregulated during infection, and investigated its role in modulating the MAPK and PI3K/AKT signaling pathways. We demonstrate that *C. trachomatis* exploits host METTL14 to activate these pathways, thereby suppressing apoptosis and promoting its intracellular reproduction.

## 2. Materials and Methods

### 2.1. Antibodies and Western Immunoblotting

The primary antibodies used in this study were as follows: Anti-GAPDH rabbit polyclonal antibody (1:5000, 10494-1-AP, Proteintech, Rosemont, IL, USA), anti-METTL14 (1:2000, 26158-1-AP, Proteintech, Rosemont, IL, USA). MAPK Family Antibody Sampler Kit (1:1000, #9926) and Phospho-MAPK Family Antibody Sampler Kit (1:1000, #9910) were purchased from Cell Signaling Technology (Danvers, MA, USA), Anti-Phospho-AKT (Ser473) rabbit monoclonal antibody (1:1000, #4060, Cell Signaling Technology, Danvers, MA, USA), anti-AKT rabbit monoclonal antibody (1:1000, #4691, Cell Signaling Technology, Danvers, MA, USA). Cells were lysed with RIPA buffer (Cwbio, Taizhou, China) supplemented with both protease and phosphatase inhibitors cocktail (Cwbio, Taizhou, China), Western blotting was performed as previously described [27]. Densitometric analysis of the Western blotting band intensities was performed using the Quantity One 4.6.2 software.

### 2.2. Cell Culture and Chlamydia Infection

HeLa 299 cells (ATCC CCL-2) were cultured in DMEM supplemented with 10% fetal bovine serum at 37 °C and 5% CO_2_. *C. trachomatis* serovar E (Bour) was propagated in HeLa 299 cells following centrifugation-assisted inoculation at a specified multiplicity of infection (MOI) of 0.8.

### 2.3. Infectious Progeny Assay

HeLa 229 cells were infected with *C. trachomatis* at a MOI following the corresponding experimental treatment. At 40 h post-infection (hpi), cells were lysed in SPG buffer with glass beads and centrifuged to remove debris. The supernatant was diluted and used to infect newly cultured HeLa cells. The secondary infected cells were fixed at 24 hpi. For each sample, at least 5 to 10 random fields were selected for immunofluorescence to count the number of chlamydial inclusions and evaluate the production of infectious progeny.

### 2.4. Total RNA Extraction and m6A Quantification

Total RNA was extracted from HeLa cells infected with *C. trachomatis* at different time points by using Eastep^®^ Super Total RNA Extraction Kit (Promega, Beijing, China) with an integrated DNase digestion step to remove residual genomic DNA. Subsequently, global m6A levels in RNA were quantified using the EpiQuik m6A RNA Methylation Quantification Kit (Colorimetric) (Epigentek, Farmingdale, NY, USA) following the manufacturer’s protocol.

### 2.5. RNA Interference

siRNAs targeting METTL14 and a negative control siRNA were purchased from Ribobio (Guangzhou, China). HeLa cells were transfected with siRNA targeting METTL14 (final concentration 20 μM) using Lipofectamine 3000 (Thermo Fisher Scientific, Waltham, MA, USA) according to the manufacturer’s instructions. At 24 to 48 h post-transfection, the cells were infected with *C. trachomatis* and harvested at the indicated time points for subsequent analyses. The siRNA sequences used in this study are listed in Supplemental Table S1.

### 2.6. m6A MeRIP-Seq

Total RNA was extracted and purified using TRIzol reagent (Invitrogen, Carlsbad, CA, USA) following the manufacturer’s protocol. The cleaved RNA fragments were incubated for 2 h at 4 °C with an m6A-specific antibody (Synaptic Systems, Goettingen, Germany) in immunoprecipitated (IP) buffer (50 mM Tris-HCl, 750 mM NaCl, and 0.5% Igepal CA-630). In addition to IP samples, input RNA (non-immunoprecipitated total RNA) was collected and sequenced in parallel as a control for normalization of transcript abundance and identification of m6A-enriched peaks. 2 × 150 bp paired-end sequencing (PE150) was conducted using an Illumina NovaSeq 6000 (LC-Bio Technology Co., Ltd., Hangzhou, China).

### 2.7. Flow Cytometry Analysis

Apoptosis rates were assessed using flow cytometry. The cells were stained with Annexin V-FITC (5 μL) and propidium iodide (PI, 4 μL) using an apoptosis detection kit (Bioscience, Shanghai, China) for 15 min. Subsequently, flow cytometry (BD Biosciences, San Jose, CA, USA) was used to determine apoptosis rates in each experimental group.

### 2.8. Statistical Analysis

All data were presented as the mean ± standard error. Comparison between two groups of data was performed using an unpaired Student’s *t*-test. Statistical analysis was performed by GraphPad Prism software 8.3.0 (La Jolla, CA, USA). *p* < 0.05 was considered statistically significant.

## 3. Results

### 3.1. m6A RNA Methylation Levels Are Altered in C. trachomatis-Infected Host Cells

**Figure 1:**
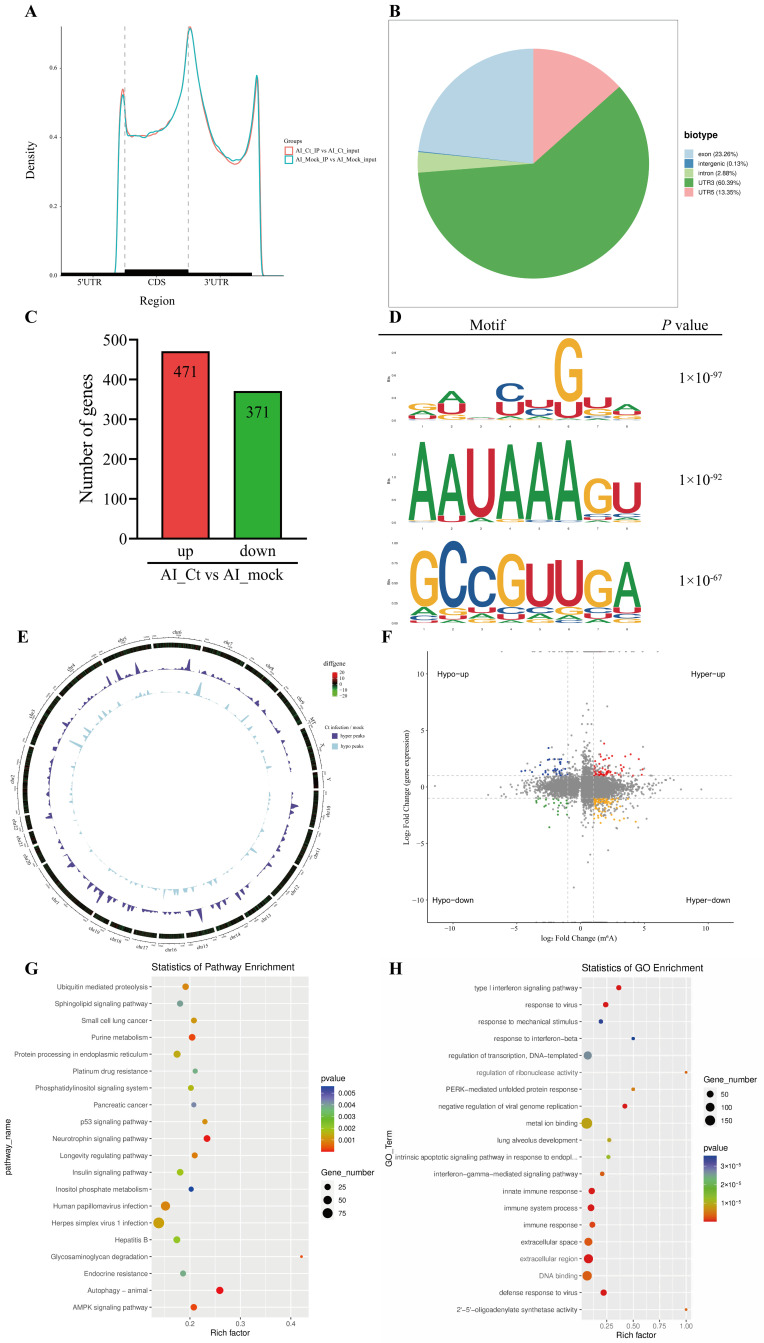
*C. trachomatis* infection modulates m6A RNA methylation and host signaling pathways. (**A**) The distribution of m6A peaks along reference mRNAs in mock-infected and *C. trachomatis*-infected HeLa299 cells at 40 hpi. (**B**) Distribution of m6A peaks across different transcript regions, including the 5′ UTR, introns, intergenic regions, exons, and 3′ UTR, in host cell transcripts. Pie charts represent the proportion of m6A peaks in uninfected and *C. trachomatis*-infected cells. (**C**) RNA-seq was conducted on the input RNA from MeRIP-seq to analyze differential mRNA expression profiles between infected and uninfected cells. (**D**) Enriched consensus m6A motifs identified by HOMER analysis from MeRIP-seq. Two independent biological replicates were used. (**E**) Circos plot illustrating the distribution of hyper- and hypomethylated m6A peaks in *C. trachomatis*-infected cells compared with uninfected cells. (**F**) Four-quadrant plot showing the distribution of genes with significant changes in both m6A modifications and gene expression in *C. trachomatis*-infected cells compared with uninfected cells. The gray dots represent genes without significant fold changes, whereas the colored dots represent genes with significant fold changes. (**G**) KEGG pathway enrichment analysis of genes with differential m6A modification. (**H**) GO enrichment analysis was conducted to characterize the functional categories of differentially expressed genes.

Previous studies have shown that pathogen infection can alter m6A modifications of host cell transcripts, thereby influencing diverse physiological and pathological processes [28]. We hypothesized that *C. trachomatis* infection could alter cellular m6A modifications, consequently affecting the expression of host genes and the activation of signaling pathways. To test this, we analyzed the abundance and distribution of m6A modifications in the RNA of HeLa cells with and without *C. trachomatis* infection. Consistent with previous studies, the identified m6A peaks were predominantly located in the internal exons and near the start of the 3′ untranslated region (3′ UTR) [29,30]. Additionally, m6A peaks were also enriched in the 5′ UTR and introns (Figure 1A,B), indicating that chlamydial infection induces widespread remodeling of m6A modifications of host transcripts. These changes may influence key processes such as gene translation, alternative splicing, mRNA stability and degradation.

To further assess transcriptional changes, RNA-seq was performed on the input fragments of MeRIP-seq to analyze the differential mRNA expression profiles between infected and uninfected controls. Compared with uninfected controls, 842 genes were differentially expressed in *C. trachomatis*-infected cells (*p* adj < 0.05, |Log_2_FC| ≥ 1), with 471 genes upregulated and 371 genes downregulated (Figure 1C). Consistent with previous studies, the known m6A modification sequence motif “DRACH” was significantly enriched in the m6A-immunopurified RNA (Figure 1D).

Genome-wide MeRIP-seq analysis identified 16,564 m6A peaks in *C. trachomatis*-infected samples (Figure 1E). Among these, 2061 peaks were significantly upregulated compared with uninfected controls (*p* adj < 0.05, Log_2_FC ≥ 1). Compared with uninfected cells, there was a significant change in the distribution of m6A-modified and differentially expressed genes in *C. trachomatis*-infected cells (Figure 1F). Kyoto Encyclopedia of Genes and Genomes (KEGG) analysis revealed that methylated genes were associated with various cellular and biological pathways, including “purine metabolism”, “p53 signaling pathway”, “autophagy”, and “AMPK signaling pathway” (Figure 1G). Gene Ontology (GO) analysis revealed that the differentially expressed genes were significantly enriched in pathways related to the “innate immune response”, “PERK-mediated unfolded protein response”, and “apoptosis” (Figure 1H). Collectively, these results demonstrate that *C. trachomatis* infection induces widespread changes in m6A modification patterns in host transcripts, potentially contributing to the regulation of host cellular responses.

### 3.2. m6A-Modified Transcripts Are Enriched in MAPK and PI3K/AKT Pathways During C. trachomatis Infection

**Figure 2:**
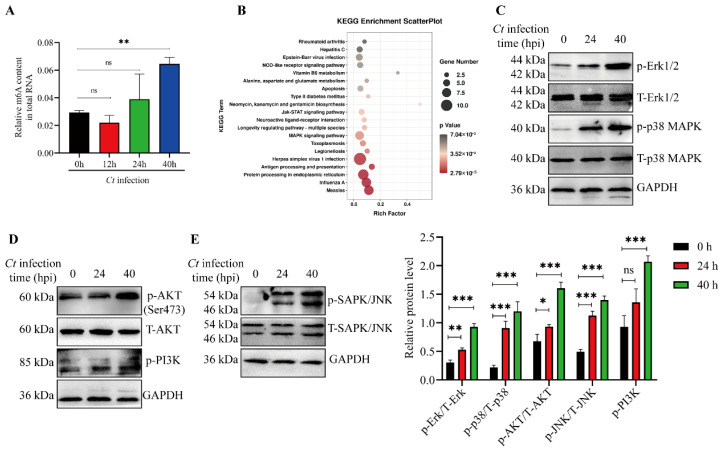
MAPK and PI3K/AKT signaling pathways involved in m6A peaks and mRNA upregulation of target genes in infected cells. (**A**) Quantification of m6A modification revealed m6A levels in total RNA of HeLa299 cells at 12, 24, and 40 hpi with *C. trachomatis*. (**B**) Pathway enrichment analysis of MeRIP-seq and RNA-seq data showed that signaling pathways were enriched among differentially m6A-modified and differentially expressed mRNA transcripts. (**C**–**E**) HeLa299 cells were infected with *C. trachomatis* for the indicated time points. Cell lysates were subjected to Western blotting to detect phosphorylated Erk1/2 (Thr202/Tyr204), p38 (Thr180/Tyr182), AKT (Ser473), PI3K (Tyr458/Tyr199), and JNK (Thr183/Tyr185), with GAPDH as a loading control. Comparison between two groups of data was performed using an unpaired Student’s *t*-test. * *p* < 0.05, ** *p* < 0.01, *** *p* < 0.001, ns: not significant (*p* > 0.05).

To investigate the effect of *C. trachomatis* infection on global m6A modification in host cells, we quantified the m6A content in total RNA isolated from HeLa cells at 12, 24, and 40 hpi. As shown in Figure 2A, m6A levels were significantly increased in infected cells compared with uninfected controls at 40 hpi.

To further identify the signaling pathways associated with genes exhibiting increased m6A modifications during *C. trachomatis* infection, we performed integrated pathway enrichment analysis using MeRIP-seq and RNA-seq data. Notably, the MAPK and PI3K/AKT (Supplemental Table S2) signaling pathways were also enriched among genes with up-regulated m6A peak and mRNA (Figure 2B), suggesting that m6A modification may contribute to the regulation of these signaling pathways during *C. trachomatis* infection. Consistently, our results confirmed that MAPK and PI3K/AKT signaling pathway were activated during *C. trachomatis* infection (Figure 2C–E). Therefore, the widespread impact of *C. trachomatis* infection on host signaling pathway transduction can be partially attributed to m6A modifications.

### 3.3. Activation of MAPK and PI3K/AKT Pathways Contributes to Apoptosis Resistance in C. trachomatis-Infected Cells

**Figure 3:**
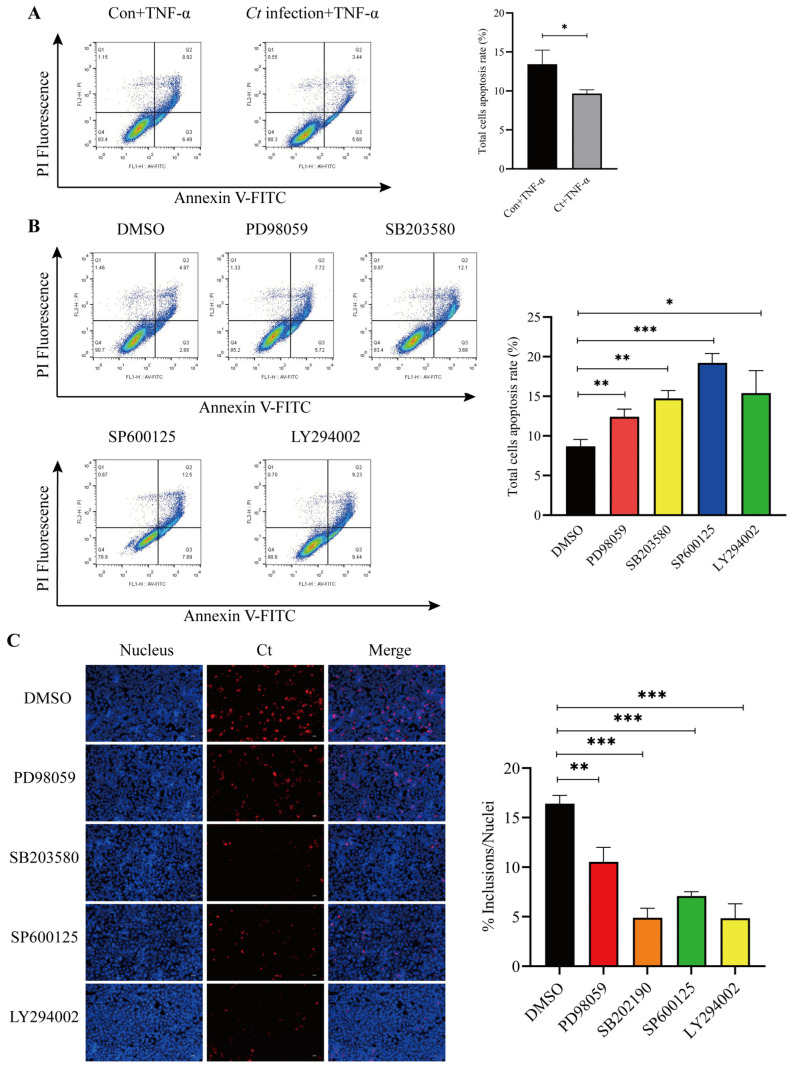
*C. trachomatis* inhibits host cell apoptosis to facilitate its intracellular reproduction by activating the MAPK and PI3K signaling pathways. (**A**) Flow cytometry analysis of TNF-α-induced apoptosis in *C. trachomatis*-infected cells at 24 hpi. (**B**) HeLa299 cells were pre-treated with specific inhibitors targeting Erk1/2 (PD98059), p38 (SB203580), JNK (SP600125), and PI3K/AKT (LY294002) for 4 h, respectively, with DMSO as a control. Cells were subsequently infected with *C. trachomatis* for 24 h, followed by flow cytometry analysis of apoptosis. (**C**) Cells were subsequently infected with *C. trachomatis* at an MOI of 0.8 and lysed at 40 hpi. Supernatants containing infectious EBs were transferred onto fresh HeLa299 monolayers for reinfection. The production of infectious progeny was assessed by immunofluorescence microscopy (200× magnification). Representative data from three independent experiments are shown. Comparison between two groups of data was performed using an unpaired Student’s *t*-test. * *p* < 0.05, ** *p* < 0.01, *** *p* < 0.001.

Previous studies have shown that *C. trachomatis* inhibits host cell apoptosis through multiple signaling pathways to sustain intracellular growth [31,32,33]. Therefore, we next investigated the roles of the MAPK and PI3K/AKT signaling pathways in *C. trachomatis*-mediated suppression of host cell apoptosis. Consistent with previous studies, *C. trachomatis* infection suppressed TNF-α-induced apoptosis (Figure 3A). Moreover, we confirmed that inhibition of Erk1/2, P38, JNK and PI3K/AKT signaling pathways significantly increased apoptosis, suggesting that *C. trachomatis* inhibits apoptosis through activation of MAPKs and PI3K/AKT signaling pathways (Figure 3B). Given that inhibition of host cell apoptosis serves as a strategy for *C. trachomatis* to facilitate its intracellular reproduction, we next investigate the role of the MAPK and PI3K/AKT signaling pathways in the intracellular reproduction of *C. trachomatis*. To address this, we selectively inhibited the Erk1/2, P38, JNK and PI3K/AKT signaling pathways using the specific inhibitors PD98059, SB203580, SP600125 and LY294002, respectively. The number of chlamydial inclusions were quantified by immunofluorescence. As shown in Figure 3C, the reproduction of *C. trachomatis* was significantly reduced following inhibition of the MAPK and PI3K signaling pathways in host cells, indicating that activation of these pathways contributes to both apoptosis resistance and efficient intracellular bacterial growth.

### 3.4. METTL14 Drives MAPK and PI3K/AKT Activation for Apoptosis Resistance

**Figure 4:**
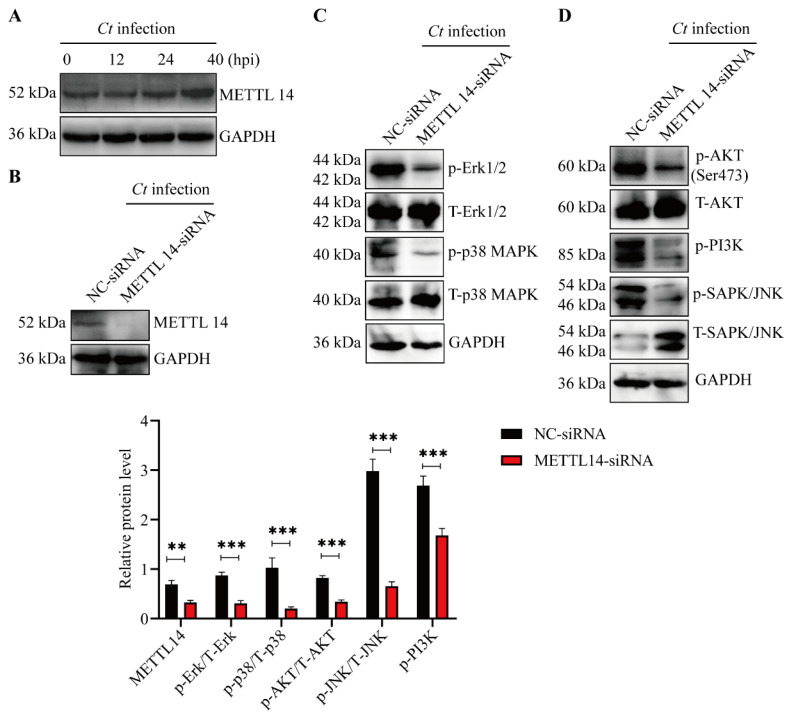
METTL14 plays a positive regulatory role in the activation of MAPK and PI3K/AKT signaling pathways. (**A**) HeLa299 cells were infected with *C. trachomatis* (MOI of 0.8) and harvested at 0, 12, 24, and 40 hpi. Western blotting analysis was conducted to examine the expression of m6A methyltransferase METTL14 using specific antibodies. GAPDH was used as a loading control. (**B**) METTL14 expression was knocked down in HeLa299 cells using METTL14-specific siRNA. NC siRNA was used as a control. Western blotting was performed to assess METTL14 protein levels. (**C**,**D**) Knockdown of the host m6A methyltransferase METTL14 suppresses the activation of Erk1/2 (**C**), p38 (**C**), PI3K/AKT (**D**), and JNK (**D**) signaling pathways. HeLa229 cells were transfected with either nontargeting control siRNA or METTL14-specific siRNA to knock down METTL14 expression. At 24 hpi, cells were infected with *C. trachomatis*. Western blotting was performed to examine the activation of MAPK (Erk1/2, p38, and JNK) and PI3K/AKT signaling pathways using the indicated antibodies. Comparison between two groups of data was performed using an unpaired Student’s *t*-test. ** *p* < 0.01, *** *p* < 0.001.

Previous studies have shown that the m6A methyltransferase METTL14 is a critical regulator of pathogen replication and immune evasion [34,35,36]. We next investigated whether *C. trachomatis* infection affects the expression of METTL14. Immunoblot analysis revealed that METTL14 expression increased at 24 and 40 hpi, indicating that the expression of this m6A-related protein is dynamically regulated during infection (Figure 4A).

To further determine whether METTL14 contributes to the activation of key host cell signaling pathways, METTL14 was silenced using siRNA, and the effects of its depletion on the MAPK and PI3K/AKT signaling pathways were evaluated (Figure 4B). We found that METTL14 knockdown significantly suppressed activation of the MAPK pathway, including Erk1/2, p38 and JNK, as well as the PI3K/AKT pathways during *C. trachomatis* infection (Figure 4C,D). Collectively, these findings demonstrate that METTL14 contributes to the activation of the MAPK and PI3K/AKT signaling pathways during *C. trachomatis* infection.

### 3.5. METTL14 Is Required for Regulating Cell Apoptosis and Chlamydial Intracellular Reproduction

**Figure 5:**
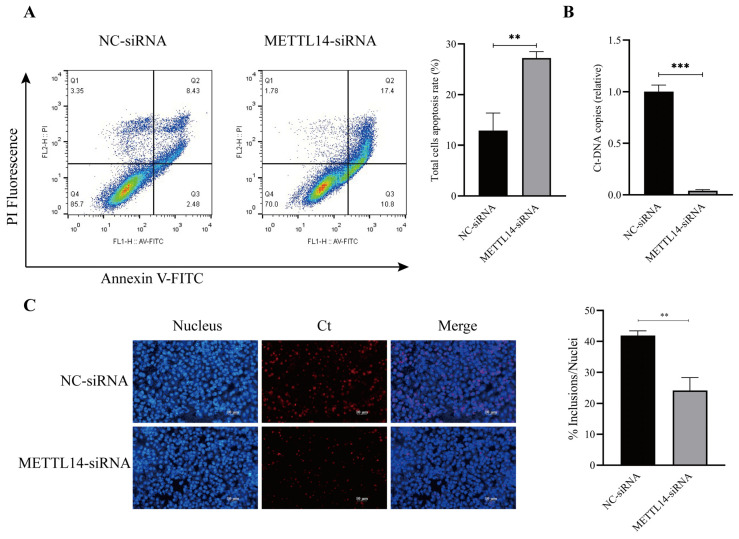
*C. trachomatis* activates the MAPK and PI3K signaling pathways via METTL14 to inhibit host cell apoptosis and facilitate its intracellular reproduction. (**A**) HeLa299 cells were transfected with either nontargeting control siRNA (NC siRNA) or METTL14 siRNA for 24 h, followed by flow cytometric analysis of apoptosis. (**B**) Total RNA was extracted from HeLa299 cells infected with *C. trachomatis*, with or without METTL14 knockdown, at the indicated time points. qPCR was used to quantify *C. trachomatis* genomic DNA copies, with GAPDH serving as an internal control. (**C**) HeLa299 cells were transfected with METTL14-specific siRNA or nontargeting control siRNA and then infected with *C. trachomatis* at an MOI of 0.8 for 40 h. Infectious progeny were harvested following cell lysis and used to infect newly cultured HeLa229 cells. At 24 hpi, cells were fixed and immunostained to visualize chlamydial inclusions. Comparison between two groups of data was performed using an unpaired Student’s *t*-test. ** *p* < 0.01, *** *p* < 0.001.

Our results indicates that METTL14 promotes activation of the PI3K/AKT and MAPK signaling pathways. Flow cytometry analysis further revealed that METTL14 depletion abrogates the anti-apoptotic effect of *C. trachomatis*, highlighting its critical role in *C. trachomatis*-mediated inhibition of host cell apoptosis (Figure 5A).

Moreover, our results demonstrate that METTL14 depletion notably decreased chlamydial DNA copy number and infectious progeny, as shown in Figure 5B,C. These results further support the notion that METTL14 suppression impairs *C. trachomatis* reproduction and infectious progeny formation within host cells. Taken together, our findings highlights the crucial role of METTL14 in both the inhibition of apoptosis and the promotion of chlamydial intracellular growth via activation of the MAPK and PI3K/AKT pathways.

## 4. Discussion

m6A is the most prevalent internal modification of mRNA at the post-transcriptional level, playing a crucial role in diverse biological functions across higher eukaryotes by modulating transcript stability, translation efficiency, and alternative splicing [37,38,39,40]. A growing number of studies have reported that m6A writers, readers, and erasers play critical roles in pathogenesis, pathogen replication, and immune evasion by modulating m6A modifications in either pathogen or host transcripts [9,41,42,43]. However, how the genome-wide dynamic pattern of m6A modification influences *C. trachomatis* infection and host cellular processes remain poorly understood.

In the present study, we identify METTL14 as a key methyltransferase component that is upregulated during *C. trachomatis* infection. This finding suggests that *C. trachomatis* infection modulates m6A-related protein expression, potentially altering mRNA methylation and facilitating chlamydial infection. Consistently, METTL14 knockdown significantly reduced *C. trachomatis* reproduction, indicating that METTL14 functions as a positive regulator of intracellular chlamydial growth. Notably, although the expression of METTL14 was significantly upregulated during infection, the expression patterns of other components of the m6A methyltransferase complex, such as METTL3 and WTAP, were not examined in this study, which warrants further investigation.

m6A modifications in specific host transcripts or signaling pathways associated with m6A modifications of target transcripts play an important role in pathogen infection, growth, and replication [34,44,45]. Considering the potential impact of m6A modifications on the lifecycle, including reproduction, of *C. trachomatis*, we utilized MeRIP and LC-MS/MS to analyze m6A-modified mRNAs in host cells post-infection. The results revealed that significant global alterations occurred in host m6A modifications following *C. trachomatis* infection. Among the cellular pathways enriched in transcripts with m6A modification changes, certain pathways were found to affect the growth of chlamydial inclusions, such as sphingomyelin signaling pathways [46], pathways that affect intracellular growth of *Chlamydia*, such as nucleotide metabolism [2], and pathways that regulate host cell death processes, including apoptosis and autophagy. We hypothesize that the effects of m6A-associated proteins on *C. trachomatis* reproduction may be attributable to the following aspects: (i) modulating host gene expression or signaling pathways that indirectly affect *C. trachomatis* reproduction; (ii) *C. trachomatis* being dependent on host-derived metabolites, including sphingomyelin, and METTL14-mediated regulation of host metabolism potentially contributing to creating a cellular environment favorable for chlamydial growth; or (iii) a combination of both.

Furthermore, METTL14 depletion abrogated *C. trachomatis*-mediated inhibition of apoptosis, suggesting that the pathogen may enhance its intracellular reproduction by upregulating METTL14. Notably, we observed that key signaling pathways involved in *C. trachomatis*-mediated suppression of apoptosis, such as the MAPK and PI3K/AKT signaling pathways, were enriched among m6A modification profiles. Consistently, we found that METTL14 functioned upstream in the activation of the MAPK and PI3K/AKT signaling pathways. Previous studies have demonstrated that these pathways play a critical role in the intracellular growth of *C. trachomatis*, while also inhibiting apoptosis [26,47,48,49]. In line with previous studies, inhibition of the MAPK and PI3K/AKT signaling pathways significantly increased apoptosis and reduced chlamydial reproduction in infected cells.

However, although our data indicate an enrichment of m6A-modified transcripts in the MAPK and PI3K/AKT pathways, the specific target mRNAs directly regulated by METTL14 remain to be identified. METTL14 may modulate the stability or translation of key signaling regulators, such as dual-specificity phosphatases or components of the PI3K pathway, which awaits further investigation in our subsequent studies. Meanwhile, in future research, rescue experiments involving the re-expression of METTL14 following gene knockdown are warranted to further determine the regulatory role of METTL14 in the MAPK and PI3K/AKT pathways. While all experiments in this study were performed in HeLa cells, we acknowledge that transformed cell lines may not fully recapitulate the epigenetic and signaling characteristics of primary epithelial cells or macrophages. Therefore, future studies validating METTL14-dependent m6A regulation and its functional effects in primary cells or tissue culture models will be essential to confirm the physiological relevance of our findings.

## 5. Conclusions

**Figure 6:**
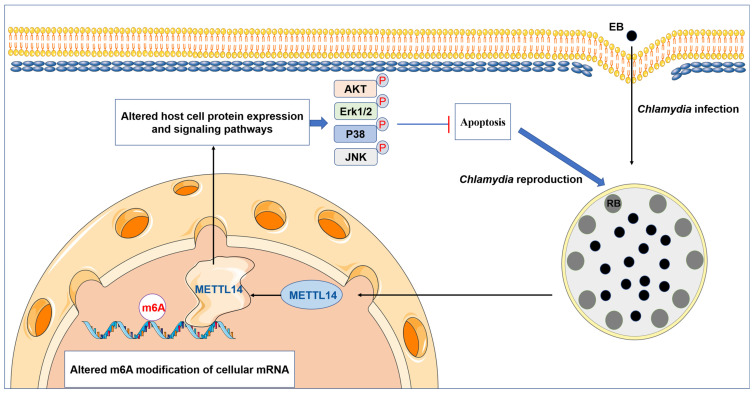
*C. trachomatis* alters key host cell signaling pathways to regulate cell death pathways and facilitate intracellular reproduction via the m6A methyltransferase METTL14. *Chlamydia* infection alters the expression levels of m6A methyltransferase METTL14, the distribution of m6A methylation motifs, and the m6A modifications on specific cellular mRNAs. METTL14, a key m6A writer enzyme, regulates *C. trachomatis* intracellular reproduction. In *C. trachomatis*-infected cells, the m6A methyltransferase METTL14 plays a crucial role in inhibiting apoptosis and promoting the intracellular reproduction of *C. trachomatis* by activating the MAPK and PI3K/AKT signaling pathways. Some elements in this figure were adapted from the SMART database (https://smart.servier.com).

In summary, our findings highlight a crucial role of m6A modifications in *C. trachomatis* reproduction and demonstrate that *C. trachomatis* exploits host METTL14 to activate the MAPK and PI3K/AKT pathways, thereby inhibiting apoptosis and facilitating its intracellular reproduction (Figure 6). This finding provides new insights into *C. trachomatis* pathogenesis and may offer potential implications for developing targeted therapies against *C. trachomatis* infection. 

## Data Availability

The raw data of the m6A-seq and RNA-seq data files were submitted to the Sequence Read Archive (SRA) database under the BioProject accession number PRJNA1036572 (https://www.ncbi.nlm.nih.gov/bioproject/PRJNA1036572; accessed on 29 April 2026).

## Supplementary Materials

The following supporting information can be downloaded at: https://www.mdpi.com/article/10.3390/microorganisms14051025/s1. Table S1: siRNA sequences for RNA interference used in this study; Table S2: KEGG enrichment analysis in Figure 2B.
