# Supplementary material for: METTL14-Mediated Inhibition of Apoptosis via the MAPK and PI3K/AKT Pathways Promotes Chlamydia trachomatis Reproduction
Source: Microorganisms. 2026 Apr 30;14(5):1025. doi: 10.3390/microorganisms14051025 (PMC13209918; doi:10.3390/microorganisms14051025)
Supplement: Supplementary file 1 [file microorganisms-14-01025-s001.zip › Supplemental Table 1 siRNA sequences for RNA interference used in this study.pdf]

Supplementary material

METTL14-Mediated Inhibition of Apoptosis via the MAPK and PI3K/AKT Pathways Promotes *Chlamydia trachomatis* Reproduction

Wenbo Lei , Yewei Yang , Yating Wen , Hongrong Wu , Zhongyu Li *

Corresponding author Zhongyu Li
Email: lzhy1023@hotmail.com

Supplemental Table 1. siRNA sequences for RNA interference used in this study.

siRNA	Ribobio number, Cat.No	Target sequence (5'-3')
genOFFTM st-h-METTL14_001	stB0012769A	CAACTACAATGCAGAAACA
siR NC	siN0000001-1-5	Proprietary
